# Excision of interferon beta-1b-associated plate-like secondary osteoma cutis

**DOI:** 10.1016/j.jdcr.2025.10.003

**Published:** 2025-10-10

**Authors:** Karenna Sandoval, David Carey

**Affiliations:** a University of New Mexico School of Medicine, Albuquerque, New Mexico; b High Desert Dermatology, Albuquerque, New Mexico

**Keywords:** Betaseron, interferon beta-1b, plate-like secondary osteoma cutis, surgical excision

## Introduction

Secondary osteoma cutis is a skin ossification disease that occurs in response to trauma, inflammation, or tumors.^1^ Surgical excision of osteoma cutis is a viable treatment method, but standard excision methods may exhibit challenges with undermining and efficient removal, particularly with larger lesions. We present a case of a successful flap approach to surgical excision of a large, plate-like secondary osteoma cutis lesion on the right lateral abdominal torso. This is a rare case of secondary osteoma cutis linked to interferon beta-1b (Betaseron) injections.

## Case presentation

**Fig 1:**
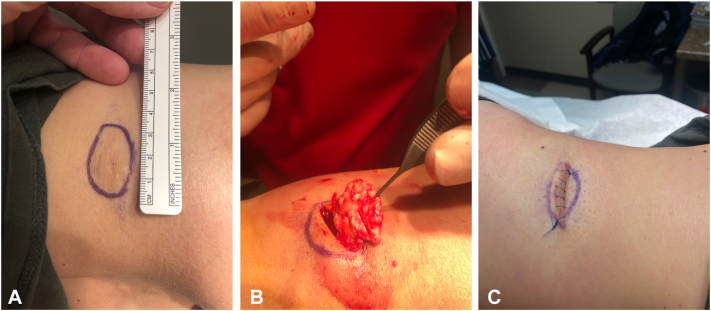
**A,** Surgical markings of plate-like secondary osteoma cutis on the patient’s left lateral abdominal torso. **B,** gross appearance of plate-like secondary osteoma cutis as it is being excised. **C,** closure using approach with a linear incision over the apex of the mass.

A 59-year-old female presented with intradermal to subcutaneous, rock-hard plates on her bilateral abdomen at the sites of interferon beta-1b injections, administered approximately 5 years prior for multiple sclerosis treatment. The plates were so large that they impinged between the iliac crest and the lowest rib with side-bending, creating considerable discomfort in her day-to-day life. Surgical excision of the most bothersome lesions was agreed upon for treatment. The initial excision of a 4.0 × 4.0 cm, rock-hard, intradermal, plate-like mass on the left lateral abdominal torso, approached with a linear incision over the apex of the mass, was successful but presented difficulties with undermining, as the free edge of the plate was sharp enough to cut the physician’s gloves, and the plate was tethered firmly and broadly across the relatively inaccessible underside (Fig 1).

**Fig 2:**
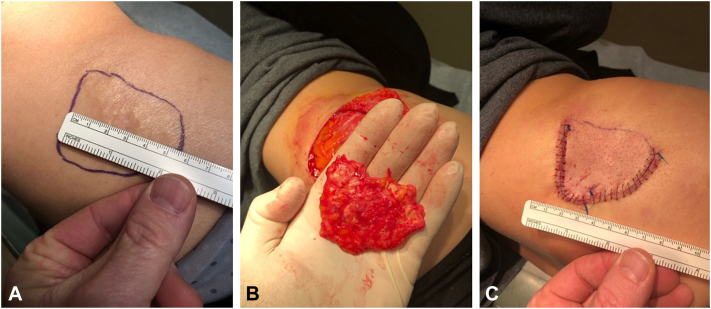
**A,** Surgical markings of plate-like secondary osteoma cutis on the patient’s right lateral abdominal torso. **B,** gross appearance of excised plate-like secondary osteoma cutis. **C,** surgical repair exhibiting flap approach.

Given the technical challenges encountered during the first excision, we modified our technique for the second, larger plate and made a curvilinear incision around the palpable free edge of the plate to create a flap that allowed access to roughly 75% of the free edge of the plate and direct visualization beneath the plate for dissection (Fig 2). After the superficial cutaneous tissues were dissected free from the top of the plate to create a flap, the flap was reflected back, and the plate was undermined with markedly less difficulty due to direct access to most of the free edge of the plate and direct visualization of the tissues beneath the plate. Closure was achieved with a combination of deep sutures of 4-0 Vicryl and superficial running sutures of 4-0 Prolene, with a final closure length of 15 cm and a flap surface area of 6.0 × 6.0 cm. No complications occurred during the procedure.

**Fig 3:**
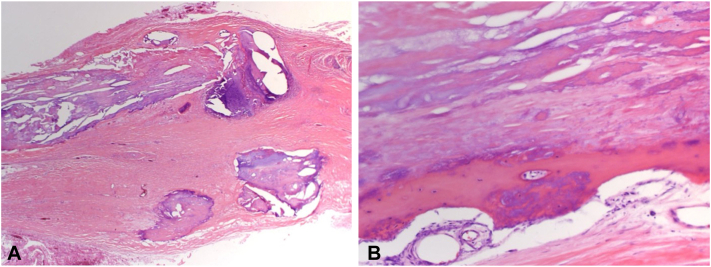
**A,** Histopathologic image of dermal to subcutaneous tissue excised from the right lateral abdominal torso with fibrosis, calcium nodules, and bone formation (hematoxylin-eosin, original magnification ×10). **B,** hematoxylin-eosin-stained section (original magnification ×40) showing mature lamellar bone formation within excised tissue consistent with osteoma cutis.

Histopathology of both specimens was consistent with osteoma cutis, revealing mature lamellar bone within the dermis, with interspersed adipose tissue (Fig 3). Altogether, these findings were consistent with the diagnosis of plate-like secondary osteoma cutis, which was successfully treated by these interventions.

## Discussion

Osteoma cutis is the aberrant extraskeletal synthesis of mature lamellar bone within the dermis and subcutaneous tissue. Fifteen percent of cases are primary, while the remaining are secondary to inflammation, trauma, or neoplastic processes.^2^ Ossification arising at sites of injection, such as in this case, has been previously reported with diphtheria-tetanus-pertussis immunization injections in children with fibrodysplasia ossificans progressiva and botulinum neurotoxin type-A injections.^3^, ^4^, ^5^ Secondary osteoma cutis has also been reported in association with basal cell carcinoma after patients had received interferon alfa-2b injections.^6^ Taken together, these cases suggest a possible association between type I interferon injections and osteoma cutis, although no mechanistic link has been elucidated to date; this remains a potential area for future research.

Plate-like osteoma cutis is a subtype of osteoma cutis, with most reported cases of plate-like osteoma cutis being primary, occurring in childhood, and related to guanine nucleotide-binding protein, alpha stimulating gene mutations. These lesions are most often located on the scalp and face.^7^ This case is a rare example of plate-like osteoma cutis that is not primary but rather connected to interferon beta-1b injections and located in a more atypical location.

Considering the successful surgical treatment of plate-like secondary osteoma cutis this case report highlights, a surgical flap approach from the edge of the plate, as described, may be an effective treatment method in the event of cases of a similar nature. Furthermore, clinicians should be made aware of this rare presentation of cutaneous ossification associated with interferon beta-1b injections used for treatment of multiple sclerosis.

## Conflicts of interest

None disclosed.
